# Concatenated ScaA and TSA56 Surface Antigen Sequences Reflect Genome-Scale Phylogeny of *Orientia tsutsugamushi*: An Analysis Including Two Genomes from Taiwan

**DOI:** 10.3390/pathogens13040299

**Published:** 2024-04-03

**Authors:** Nicholas T. Minahan, Tsai-Ying Yen, Yue-Liang Leon Guo, Pei-Yun Shu, Kun-Hsien Tsai

**Affiliations:** 1 Institute of Environmental and Occupational Health Sciences, College of Public Health, National Taiwan University, Taipei 100025, Taiwan; f05841021@ntu.edu.tw (N.T.M.); leonguo@ntu.edu.tw (Y.-L.L.G.); 2 Centers for Diagnostics and Vaccine Development, Centers for Disease Control, Ministry of Health and Welfare, Taipei 115210, Taiwan; yenty@cdc.gov.tw (T.-Y.Y.); cdcpyshu@gmail.com (P.-Y.S.); 3 Department of Environmental and Occupational Medicine, National Taiwan University (NTU) College of Medicine and NTU Hospital, Taipei 100025, Taiwan; 4 Global Health Program, College of Public Health, National Taiwan University, Taipei 100025, Taiwan

**Keywords:** genotyping, phylogenetics, scrub typhus, surface cell antigens

## Abstract

*Orientia tsutsugamushi* is an obligate intracellular bacterium associated with trombiculid mites and is the causative agent of scrub typhus, a life-threatening febrile disease. Strain typing of *O. tsutsugamushi* is based on its immunodominant surface antigen, 56-kDa type-specific antigen (TSA56). However, TSA56 gene sequence-based phylogenetic analysis is only partially congruent with core genome-based phylogenetic analysis. Thus, this study investigated whether concatenated surface antigen sequences, including surface cell antigen (Sca) proteins, can reflect the genome-scale phylogeny of *O. tsutsugamushi*. Complete genomes were obtained for two common *O. tsutsugamushi* strains in Taiwan, TW-1 and TW-22, and the core genome/proteome was identified for 11 *O. tsutsugamushi* strains. Phylogenetic analysis was performed using maximum likelihood (ML) and neighbor-joining (NJ) methods, and the congruence between trees was assessed using a quartet similarity measure. Phylogenetic analysis based on 691 concatenated core protein sequences produced identical tree topologies with ML and NJ methods. Among TSA56 and core Sca proteins (ScaA, ScaC, ScaD, and ScaE), TSA56 trees were most similar to the core protein tree, and ScaA trees were the least similar. However, concatenated ScaA and TSA56 sequences produced trees that were highly similar to the core protein tree, the NJ tree being more similar. Strain-level characterization of *O. tsutsugamushi* may be improved by coanalyzing ScaA and TSA56 sequences, which are also important targets for their combined immunogenicity.

## 1. Introduction

*Orientia tsutsugamushi* (Rickettsiales: Rickettsiaceae) is an obligate intracellular alphaproteobacterium associated with trombiculid mites [1] and is the causative agent of scrub typhus, an acute febrile disease endemic in the Asia–Pacific region [2,3]. In Taiwan, scrub typhus is the most prevalent endemic vector-borne disease, with ~400 cases confirmed annually [4]. Though readily treated with doxycycline and other antibiotics [5,6], scrub typhus remains commonly fatal in India [7] and Southeast Asia [8]. Vaccine development for scrub typhus has been challenging due to natural immunity directed against highly variable surface antigens [9], including immunodominant 56-kDa type-specific antigen (TSA56) [10], which binds to fibronectin and exploits integrin-mediated signaling for intracellular invasion [11].

Strain typing of *O. tsutsugamushi* was historically performed serologically, initially using complement fixation [12] and, later, indirect immunofluorescence assay (IFA) [13], which has remained the gold standard for serological confirmation [14], typically using whole-cell antigens of three prototype strains: Gilliam, Karp, and Kato [15]. Hybridoma technology facilitated more precise antigenic separation of isolates using monoclonal antibodies (mAbs) directed against TSA56 [10,16,17,18,19,20], although serotyping was supplanted by TSA56 gene sequence (*tsa56*)-based genotyping in the late 1990s [21]. In the 2000s, divergent strains were identified based on *tsa56* sequences among isolates in Thailand [22] and Taiwan [23,24]. Routine isolation of *O. tsutsugamushi* from acute blood specimens of scrub typhus patients has been performed at the Taiwan Centers for Disease Control (Taiwan CDC) since 2006, with complete *tsa56* sequences obtained for 545 isolates through 2016 [25]. Among 36 *tsa56* phylotypes, *O. tsutsugamushi* strains TW-1 and TW-22 are most common throughout the main and offshore islands of Taiwan, representing ~54% of clinical isolates [25]. Globally, culture-independent PCR amplification has increasingly been applied for strain characterization of *O. tsutsugamushi* targeting partial *tsa56* fragments [26,27]. At least 17 discrete *tsa56* genotypes of *O. tsutsugamushi* have been identified [28], but few complete genome sequences have been obtained [29,30,31], in part due to difficulty in sequencing its repeat-rich genome [32]. The genome of *O. tsutsugamushi* is a circular chromosome that is 1.9–2.5 Mbp in size (30.2–30.8% GC content) with 1949–2560 protein-coding sequences (CDSs) and 416–566 pseudogenes (19–24% of CDSs) [33] and is devoid of plasmids [30] but possesses >70 copies of Rickettsiales amplified genetic element, an integrative and conjugative element that, except for one copy in Kato and three copies in Gilliam, is incomplete with the pseudogenization of genes required for horizontal transfer [34].

It was not until 1995 that *O. tsutsugamushi* was demarcated from *Rickettsia* spp. with recognition of its distinct cellular envelope, surface antigens, growth characteristics, and divergent 16S rRNA gene (*rrs*) sequences [35,36]. Today, the genetic basis of these differences has largely been elucidated [37], but the function of some surface antigens remains unknown. *Orientia* and *Rickettsia* have distinct autotransporter domain-containing surface cell antigen (Sca) proteins with secreted or surface-displayed passenger domains [38]. Sca genes are among those used for the speciation of *Rickettsia* (superseded by genome-based similarity measures [39]), based on pairwise nucleotide sequence homologies of *sca0* (*ompA*), *sca4* (gene D), and *sca5* (*ompB*), in addition to *rrs* and *gltA* (citrate synthase gene) [40]. Some Sca proteins are absent in certain *Rickettsia* clades (e.g., Sca0 in the typhus group) [40], and such variation is observed at the strain level for *O. tsutsugamushi* [41]. Intact genes encoding ScaA, ScaC, ScaD, and ScaE are present in all complete *O. tsutsugamushi* genomes sequenced to date (i.e., core genes), while genes encoding ScaB and ScaF are only present in a subset of strains (i.e., accessory genes). Among core Sca proteins, only ScaA and ScaC are of known function and are involved in cellular attachment, binding to an isoform of the mixed-lineage leukemia 5 protein [42] and fibronectin [43], respectively. Like TSA56, Sca proteins (particularly ScaA) are immunogenic [44] yet reveal different phylogenetic relationships [41].

Initial core genome-based phylogenetic analysis of *O. tsutsugamushi* revealed incongruencies with analysis based on *tsa56* and other targets [31]. Given that whole-genome sequencing is not widely available for strain-level characterization of *O. tsutsugamushi*, it is desirable to improve upon single locus genotyping. Thus, this study investigated whether concatenated surface antigen sequences, including Sca proteins, can reflect the genome-scale phylogeny of *O. tsutsugamushi*.

## 2. Materials and Methods

### 2.1. Cultivation, Purification, and Genomic DNA Isolation

Two representative *O. tsutsugamushi* strains in Taiwan, TW-1 and TW-22, were selected for whole-genome sequencing (WGS). Clinical *O. tsutsugamushi* isolates LC0708a (TW-1) and KHC0708a (TW-22) (originally reported in [25]) were recovered in mouse (*Mus musculus*) fibroblast-like L929 cells (BCRC # RM60091) from frozen stocks at the Taiwan CDC Laboratory of Vector-borne Viral and Rickettsial Diseases. LC0708a was isolated from a 19-year-old male who presented with fever and rashes in Lienchiang County (Matsu Islands) in August 2007, and KHC0708a was isolated from a 45-year-old female who presented with fever, headache, malaise, lymphadenopathy, and an eschar in Kaohsiung City in August 2007. L929 cells were maintained in a 75 cm^2^ (T75) flask using MEM (Gibco, Grand Island, NY, USA) supplemented with 4% fetal bovine serum (FBS) (Gibco) and 1% Antibiotic-Antimycotic (Gibco) at 37 °C with 5% CO_2_. Frozen *O. tsutsugamushi* stocks (0.5 mL) were rapidly thawed, resuspended to disrupt host cells, and used to inoculate L929 cells at ~60% confluence in a 25 cm^2^ flask with a small volume of serum-free MEM and incubated at 32 °C with 5% CO_2_ for 60–90 min, followed by the addition of MEM containing 2% FBS and 1% Antibiotic-Antimycotic. Media was changed within 24 h and again within 72 h (on day 3 or 4, depending on cell health). Bacterial load was monitored with semiquantification of *O. tsutsugamushi* DNA extracted from culture supernatant with SYBR Green-based quantitative PCR (qPCR) targeting a 120 bp fragment of the single-copy 47-kDa gene (*tsa47*) [45], examining changes in cycle threshold (C_T_) values. This assay was performed in 20 µL reactions with 1X KAPA SYBR FAST qPCR Master Mix (Roche, Basel, Switzerland), 0.2 µM of each primer (synthesized by Mission Biotech, Taipei, Taiwan), and 2 µL of DNA template or water (no template control), and qPCR was performed using a MyiQ2 thermal cycler (Bio-Rad, Hercules, CA, USA) at 95 °C for 3 min and 40 cycles of 95 °C for 3 s and 60 °C for 20 s followed by a dissociation curve analysis from 65 °C to 95 °C with 0.5 °C increments. Once bacterial growth reached the late exponential phase, cells were harvested for preservation at −80 °C via gentle scraping, and remaining cells were disrupted with 0.5 mm glass beads to release intracellular *O. tsutsugamushi* for passage. Briefly, the cell suspension was diluted in serum-free MEM (1:10 to 1:20 based on relative load) to infect fresh L929 cells as before, except in a T75 flask. This process was repeated until passage 8, upon which 5 to 8 flasks were inoculated to harvest *O. tsutsugamushi* for purification and genomic DNA extraction.

Filter purification and DNA isolation were performed using a similar approach to Batty et al. [31]. Once *O. tsutsugamushi* growth was in the stationary phase, host cells were disrupted by gently agitating the flask containing 0.5 mm glass beads with a small volume of spent media, and the lysate, recovered using spent media, was filtered through a 2 µm pore size Puradisc 25 syringe filter (Whatman, Maidstone, UK). Filtered *O. tsutsugamushi* cells were pelleted at 14,000× *g* for 10 min, the supernatant was discarded, and cells were resuspended using 380 µL RDD Buffer (Qiagen, Hilden, Germany) and divided into two equal volumes for further processing. Residual host cell genomic DNA was depleted by adding 2.5 µL Benzonase nuclease (Qiagen) to each tube, with incubation in a 37 °C water bath for 30 min, followed by enzyme inactivation with the addition of 20 µL Proteinase K (Qiagen) and incubation at 56 °C for 30 min. *O. tsutsugamushi* cells were pelleted as before, the supernatant was discarded, and cells were resuspended in Dulbecco’s phosphate-buffered saline. DNA was isolated using the DNeasy Blood and Tissue Kit (Qiagen) following the manufacturer’s protocol, proceeding with the addition of 20 µL Proteinase K and 200 µL Buffer AL (Qiagen), gentle mixing, and incubation at 56 °C for 10 min. DNA was eluted using 100 µL 10 mM Tris-Cl (pH 8.5) per column, stored at 4 °C, and quantified using a Qubit fluorometer (dsDNA HS Assay Kit; Invitrogen, Waltham, MA, USA) and Fragment Analyzer 5200 (DNF-464 Kit; Agilent, Santa Clara, CA, USA).

### 2.2. Quantitative PCR

SYBR Green-based qPCR targeting *tsa47* and a 108 bp fragment of the single-copy mouse adipsin gene (*cfd*) [46] was performed to evaluate the depletion of host cell genomic DNA. Triplicate 20 µL reactions were performed, each containing 1X iTaq Universal SYBR Green Supermix (Bio-Rad), 0.5 µM of each primer, and 2 µL of DNA template or water, and qPCR was performed using an ABI 7300 thermal cycler (Applied Biosystems, Foster City, CA, USA) at 95 °C for 5 min and 40 cycles of 95 °C for 15 s and 60 °C for 60 s followed by a dissociation curve analysis (system default). Copy number was determined based on calibration curves constructed using pCR2.1-TOPO vector (Thermo Fisher Scientific, Waltham, MA, USA) containing target gene fragments (*tsa47* from Taiwan CDC Karp and *cfd* from L929) with 10^9^ to 10^4^ and 10^7^ to 10^2^ copies per reaction (serially diluted in 10-fold increments) for *tsa47* and *cfd* assays, respectively. Linear regression was performed in R 4.3.0 (https://www.r-project.org/, accessed on 25 April 2023), and ggpubr 0.6.0 [47] was used for data visualization. The percentage of residual host cell genomic DNA was calculated referencing a genome size of 2.7 Gbp for *M. musculus* (reference assembly GRCm39; RefSeq GCF_000001635.27) and 2 Mbp for *O. tsutsugamushi* (reference assembly Ikeda; RefSeq GCF_000010205.1).

### 2.3. Whole Genome Sequencing, Assembly, and Annotation

WGS was performed by Genomics Bioscience and Technology Co., Ltd. (New Taipei City, Taiwan) using the PacBio Sequel sequencing platform (Pacific Biosciences, Menlo Park, CA, USA). Briefly, genomic DNA was sheared using a g-TUBE (Covaris, Woburn, MA, USA) and purified with AMPure PB beads (Beckman Coulter, Brea, CA, USA) for ~10 kbp libraries. SMRTbell libraries were sequenced using a SMRT Cell 1M v3 (Sequel Sequencing Kit 3.0; Pacific Biosciences).

Following sequencing, HiFi reads were generated from subreads using ccs 6.4.0 [48], and reads that mapped to the *M. musculus* genome using minimap2 2.26-r1175 [49] were removed with SAMtools 1.15.1 [50]. Additionally, HiFi reads containing residual PacBio adapter sequences were identified and removed using ShortRead 1.56.1 [51] and Biostrings 2.67.2 [52]. De novo assembly was performed using filtered HiFi reads using hifiasm 0.19.5-r587 [53] with five rounds of overlap/error correction and assembly cleaning (-r 5 -a 5). If a circular contig was not obtained, Circlator 1.5.5 [54] was used with Canu 1.4 [55]; otherwise, only the “fixstart” function in Circlator was used to reorient genomes to start with *dnaA*. Filtered HiFi reads were aligned to draft assemblies using BWA 0.7.17-r1188 [56] and used as the input to Pilon 1.24 [57] for polishing and determining coverage. Annotation was performed using the NCBI Prokaryotic Genome Annotation Pipeline (PGAP) 2023-05-17.build6771 [33,58]. Circular chromosome maps were constructed using the Proksee web server [59]. Annotated genomes were submitted to NCBI GenBank (BioProject Accession Number PRJNA987430).

### 2.4. Phylogenetic Analysis

Complete genomes of nine *O. tsutsugamushi* strains (Boryong, Ikeda, Gilliam, Karp, Kato, TA686, UT76, UT176, and Wuj/2014), listed in Table 1 with their country of origin and year of isolation, were retrieved from GenBank [60]. Interstrain variation for each surface antigen was examined with global alignment of amino acid sequences in Jalview 2.11.2.7 [61] with “pairwise alignment”, which uses the BLOSUM62 scoring matrix. The M1CR0B1AL1Z3R web server [62] was used to identify the core genome/proteome with the minimal percent of identity set to 70%. Multiple sequence alignment (MSA) was performed using MAFFT [63] as implemented via M1CR0B1AL1Z3R, surface antigen MSAs were concatenated using MEGA11 [64], and positions containing gaps were removed using TrimAl 1.4.rev15 [65]. Maximum likelihood (ML) trees were constructed using RAxML-NG 1.2.0 [66] with the JTT + I + G4 + F model, 20 random and 20 parsimony starting trees, and 1000 bootstrap replicates. Neighbor-joining (NJ) [67] trees were constructed using MEGA11 with the JTT amino acid substitution model [68] with a discrete gamma distribution with four rate categories and 1000 bootstrap replicates [69]. Phylogenetic trees were visualized using MEGA11 and phytools 1.5.1 [70], and Boryong was used to root trees. Congruence between trees was evaluated using the “SimilarityToReference” function implemented in Quartet 1.2.5 [71,72].

## 3. Results

### 3.1. Complete Genomes of O. tsutsugamushi Strains TW-1 and TW-22

Both strains had similar growth kinetics after repeated passages in L929 (Figure S1). Isolated DNA consisted of large fragments concentrated at 53.3 kbp for TW-1 and 48.5 kbp for TW-22 with 0.03% and 0.04% L929 genomic DNA content, respectively, and SMRTbell library fragments were concentrated at 10.5 kbp for TW-1 and 12.7 kbp for TW-22. TW-1 yielded 1.9 × 10^6^ subreads (5.1 × 10^9^ bases) and generated 7.8 × 10^4^ HiFi reads (2.4 × 10^8^ bases) with a mean length of 3025 bp, and 0.22% of HiFi reads (0.15% of bases) mapped to the mouse genome. TW-22 yielded 1.1 × 10^6^ subreads (4.8 × 10^9^ bases) and generated 5.2 × 10^4^ HiFi reads (2.6 × 10^8^ bases) with a mean length of 5079 bp, and 0.28% of HiFi reads (0.14% of bases) mapped to the mouse genome. Residual PacBio adapter sequences were detected in 10 and 8 HiFi reads for TW-1 and TW-22, respectively. Filtered HiFi reads used for assembly are summarized in Figure S2. De novo assembly with hifiasm produced a single linear contig 2,014,300 bp in length for TW-1, and a circular contig 2,008,429 bp in length with 30.49% GC content was obtained with Circlator (Figure S3). A circular contig 2,044,475 bp in length with 30.49% GC content was obtained for TW-22 from hifiasm (Figure S4). Pilon confirmed bases in both assemblies using alignments of filtered HiFi reads with coverage of 116 (minimum depth of 12) for TW-1 and 128 (minimum depth of 13) for TW-22. A total of 2067 genes were annotated in TW-1 (2027 CDSs with 400 pseudogenes) (Figure S3) and 2192 genes in TW-22 (including 2152 CDSs with 414 pseudogenes) (Figure S4), and both genomes had 40 RNA genes (including 3 rRNAs, 34 tRNAs, and 3 other ncRNAs). These features are comparable with previously complete *O. tsutsugamushi* genomes (Table 1). Among 1627 intact CDSs in TW-1, 354 were hypothetical proteins (HPs), and there were 400 HPs among 1738 intact CDSs in TW-22. TSA56 gene sequences were 100% identical to their reference accessions of MW495332 (1608/1608) for TW-1 and MW495697 (1575/1575) for TW-22.

### 3.2. Core Genome Phylogeny of O. tsutsugamushi

**Figure 1:**
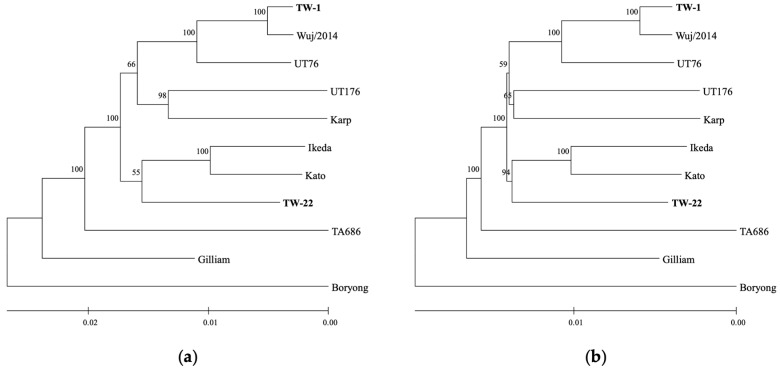
Phylogenetic analysis of 11 *Orientia tsutsugamushi* strains based on 691 concatenated core protein sequences (235,464 positions without gaps) based on (**a**) maximum likelihood with RAxML-NG v1.2.0 [66], performed using the JTT + I + G4 + F model substitution (tree with the highest log-likelihood is shown) and (**b**) neighbor-joining with MEGA11 [64] based on evolutionary distances computed using the JTT matrix with 4 discrete gamma categories (optimal tree is shown). Scale branch lengths represent the number of amino acid substitutions per site, and the percentage of replicate trees in which the associated taxa clustered together in 1000 bootstrap replicates are shown above the branches.

A core of 691 CDSs was identified among 11 *O. tsutsugamushi* strains, resulting in a concatenated amino acid alignment with 243,706 positions, which was reduced to 235,464 positions with 91.91% invariant sites after removal of positions containing gaps. Phylogenetic analysis based on 691 concatenated core protein sequences (235,464 positions) produced identical tree topologies with ML and NJ methods (Figure 1). TW-1 and TW-22 were in separate clades with Karp and Kato, respectively; TW-1 most related to Wuj/2014, UT76, and then UT176 and Karp; and TW-22 related to Ikeda and Kato, while TA686 and Gilliam were on separate ancestral branches with Boryong forming an outgroup.

### 3.3. Surface Antigen-Based Phylogeny of O. tsutsugamushi

TSA56 and four Sca proteins (ScaA, ScaC, ScaD, and ScaE) were included in the core proteome, while ScaB was only identified in Boryong with two copies, and ScaF was only identified in Karp and TA686 (99.38% identity; alignment = 645 amino acids (aa); score = 32,750 bits) (Table S1; see Table S2 for locus tags). TSA56 had a maximum pairwise identity of 99.63% between TW-1 and Wuj/2014 (alignment = 535 aa; score = 27,110 bits) and a minimum pairwise identity of 68.93% between Kato and Boryong (alignment = 544 aa; score = 17,630 bits) (Table S1). ScaA had a maximum pairwise identity of 89.16% between TW-1 and Wuj/2014 (alignment = 1532 aa; score = 70,100 bits) and a minimum pairwise identity of 73.52% between TA686 and Ikeda (alignment = 1522 aa; score = 55,160 bits). ScaC was identical in TW-1 and Wuj/2014 and had a minimum pairwise identity of 86.35% between Ikeda and Karp (alignment = 520 aa; score = 23,030 bits). ScaD had a maximum pairwise identity of 95.41% between UT176 and Kato (alignment = 872 aa; score = 42,340 bits) and a minimum pairwise identity of 63.53% between Gilliam and Boryong (alignment = 998 aa; score = 26,620 bits) (Table S1). ScaE had a maximum pairwise identity of 99.73% between TW-1 and Wuj/2014 (alignment = 749 aa; score = 38,250 bits) and a minimum pairwise identity of 71.10% between Kato and Boryong (alignment = 775 aa; score = 25,470 bits) (Table S1). After the removal of positions containing gaps from MSAs, TSA56 had 498 positions (55.62% invariant), ScaA had 1412 positions (57.58% invariant), ScaC had 517 positions (74.66% invariant), ScaD had 676 positions (77.66% invariant), and ScaE had 711 positions (64.70% invariant).

**Figure 2:**
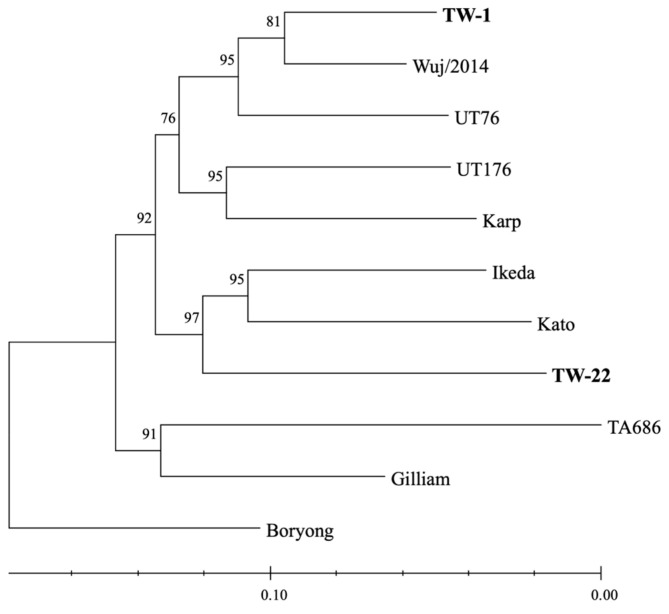
Neighbor-joining-based phylogenetic analysis of 11 *Orientia tsutsugamushi* strains based on concatenated ScaA and TSA56 amino acid sequences (1910 positions without gaps) with MEGA11 [64] based on evolutionary distances computed using the JTT matrix with 4 discrete gamma categories. The optimal tree is shown (scale branch lengths represent the number of amino acid substitutions per site), and the percentage of replicate trees in which the associated taxa clustered together in 1000 bootstrap replicates are shown above the branches.

Among individual surface antigens, TSA56 trees had the highest congruence with the core tree, while ScaA trees had the lowest (Table 2; Figures S5 and S6). Concatenated ScaA and TSA56 trees were highly congruent with the core tree, and the NJ tree had higher congruence than the ML tree (Table 2; Figure 2 and Figure S7). Concatenated ScaC and TSA56 produced an ML tree with similar congruence to the core tree as the ML tree for TSA56 alone, though with a different topology, and an NJ tree with lower congruence (Table 2; Figures S7 and S8). Concatenation of ScaD or ScaE with TSA56 also produced trees with higher congruence to the core tree than TSA56 trees (Table 2; Figures S7 and S8).

## 4. Discussion

This study found that phylogenetic analysis based on concatenated ScaA and TSA56 sequences produces trees highly similar to core protein-based phylogeny despite a >100-fold difference in the number of aligned amino acid positions analyzed. TSA56-based trees were most similar to the core tree among the surface antigens examined in this study but still had many incongruencies between phylogenies, and ScaA-based trees were highly dissimilar. This suggests that ScaA possesses phylogenetically informative sites subject to different evolutionary pressures than TSA56, which may be clarified by characterizing their protein–protein interactions. Sca proteins translocate via type V secretion [38], and while this system has not been characterized for *Orientia*, it likely involves a β-barrel assembly machine complex and other periplasmic chaperons similar to *Rickettsia* [73]. The translocation mechanism of TSA56 has not been described, but it possesses an N-terminal signal peptide that appears to be cleaved [10]. ScaA requires a conserved block (CB2, Boryong aa 843 to 875) and involves its flanking regions (fragments F4 and F5, Boryong aa 607 to 994 and 867 to 1241) for attachment, with F5 exhibiting the highest immunogenicity (i.e., anti-ScaA IgG titer) [42]. TSA56 primarily binds fibronectin at its surface-exposed antigen domain III and adjacent C-terminal region (Boryong aa 312 to 341) [11], which is relatively conserved [74] and may work in concert with ScaC [43]. TSA56 produces a robust humoral response [75] with multiple B-cell epitopes [10,76]; as such, recombinant protein-based enzyme-linked immunosorbent assays have been developed detecting anti-TSA56 antibodies for clinical diagnosis [77,78,79]. Neutralizing antibodies are important for protective immunity, but cellular immunity is also necessary to mount an effective immune response against intracellular pathogens [80]. TSA56 elicits limited T-cell responses compared to other immunoprevalent antigens, including TSA22 [81], which remains uncharacterized, and TSA47 [75], a periplasmic serine protease involved in cellular exit [82]. Notably, coimmunization of mice with ScaA and TSA56 provided enhanced protection against lethal challenge with heterologous strains compared to immunization with either antigen alone [44]; however, even in natural infection, ScaA- and TSA56-directed B- and T-cell immunity rapidly declines after one year [80], though multidose vaccines may overcome this shortcoming. Recently, nanoparticle vaccines have demonstrated enhanced immunogenicity with ScaA, TSA56, and TSA47 subunits, with enhanced protection provided by dual-layered antigen nanoparticles [83,84]. In the future, enhanced heterologous protection could be provided via nanoparticle vaccines that combine antigens from multiple strains, similar to what has been implemented for influenza viruses [85] and, importantly, may be tailored for different geographic regions. To this end, the determination of ScaA and TSA56 sequences is indispensable to identify representative antigen sequences for vaccine development.

Phylogenetic trees produced using ScaA and TSA56 were not perfectly congruent with the core protein-based tree. Gilliam and TA686 were not placed on separate branches due to their high phylogenetic relatedness for ScaA. Additionally, using ML, TW-1 and Wuj/2014 were placed on different branches, though poorly supported with bootstrap replicates (<50%), whereas NJ, which is computationally much less intensive than ML, produced a topology that was more similar to the core protein-based tree with a higher level of bootstrap confidence across nodes (>75%). Even so, for core trees, both methods had nodes with low bootstrap support (<75%) but only consistently for the node separating the group containing TW-1, Wuj/2014, and UT76 and the group containing UT176 and Karp. This could be due to geographic relatedness between Thai strains UT76 and UT176 for CDSs other than ScaA and TSA56. Boryong formed an outgroup in core phylogenetic analyses and was also found to be ancestral in a preliminary phylogenetic analysis for *Orientia* spp. based on core CDSs with the inclusion of a partial assembly of *Orientia chuto* Dubai (RefSeq GCF_000964595.1) (findings not shown). ScaB was only identified in Boryong, which has been implicated in adherence to and invasion of nonphagocytic cells [86]. ScaB has also been detected in TA686 [86] but has a gene sequence below the minimum identity threshold used to identify core CDSs in this study, which was relaxed from 80%, as used in the previous core phylogenetic analysis of *O. tsutsugamushi* with 657 core genes [31], to 70% in order to include *tsa56* as a core CDS. The expression of this core proteome still needs to be verified, including in its natural host, while 599 of the previous 657 core genes were found to be transcribed in Karp and UT176 infecting human umbilic vein endothelial cells [87]. Among other Sca proteins, ScaF was only identified in TA686 and Karp, which were clearly separated in the core tree, suggesting that ScaF has evolved multiple times, though its function remains unknown. TA686 and Karp also possessed similar ScaC, and whether ScaF is also involved in adherence in these strains should be determined. Most studies on adherence have been conducted using nonphagocytic cells [11,42,43,86], but *O. tsutsugamushi* also infects monocytes and antigen-presenting cells at the site of inoculation [88]. It has yet to be determined whether variation in core Sca proteins or the presence of accessory Sca proteins control cellular tropism, which could explain variation in strain-level virulence among mice strains and nonhuman primates [89]. In systemic infection, *O. tsutsugamushi* infects endothelial cells with the highest bacterial loads found in the lungs [90] and interstitial pneumonitis is commonly observed in severe cases which can progress to fatal acute respiratory distress syndrome [91,92], with macrophages playing a key role in pathogenesis [93].

Phylogenetic clustering did not consistently correspond with geographic origin for the 11 strains examined in this study. TW-1 was highly similar to Wuj/2014, which was isolated in Zhejiang, China (near Taiwan). TW-1 is the predominant strain isolated from scrub typhus patients in the offshore islands near China (Kinmen, Matsu, and Penghu) [25]. TW-22 was most related to Ikeda and Kato, isolated in Japan to the north. However, TW-22 is predominantly isolated in southern Taiwan [25], which has a tropical climate. Ancestral to the aforementioned strains, TA686 and Gilliam were isolated in neighboring countries in Southeast Asia (Thailand and Burma), but TA686 was not found to cluster with other Thai strains (UT76 and UT176) in the Karp clade. Phylogenetic placement of Boryong (isolated in Korea), ancestral to TA686 and Gilliam, further obfuscates the phylogeographic picture. Thus, additional genomes of geographically diverse isolates (with adequate representation for each *tsa56* genotype) are needed to clarify the phylogeography of *O. tsutsugamushi*. To this end, an effort should be made to obtain complete genomes for all described *tsa56* genotypes in Taiwan. Studies are also needed to investigate the mite fauna of migratory birds, which have long been thought to play an important role in the dissemination of *O. tsutsugamushi* [94] and have been implicated in the spread of other acarids [95]. There are at least 47 trombiculid mite species throughout Taiwan [96], but the association between mite species and *O. tsutsugamushi* strains remains unclear, and mite host–*O. tsutsugamushi* interactions remain poorly characterized. A single mite colony may be coinfected with *O. tsutsugamushi* [97], facilitating intragenic recombination [28]. Competition with cocirculating Rickettsiaceae also needs to be clarified; for example, *Rickettsia felis*-like organisms that have been found to infect *Leptotrombidium deliense* in Taiwan [98]. Globally, no complete genome sequences have been made publicly available for recently described divergent *Orientia* spp., including *Orientia chuto* (endemic in the Middle East) [99] and *Candidatus* Orientia chiloensis (endemic in South America) [100], and no criteria have been established for delineation of novel *Orientia* species. These taxa appear to be ancestral to *O. tsutsugamushi* and may shed light on the evolutionary origins of Sca proteins in *Orientia*, which has yet to be elucidated [73].

There are still methodological limitations in the ability to amplify and sequence complete *tsa56* and *scaA*, as they are large (1.6 kbp and 4.3 to 4.6 kbp, respectively), and this is particularly challenging for culture-independent studies yielding small amounts of fragmented DNA. Nonetheless, long-range high-fidelity PCR can be used to amplify complete or nearly complete *tsa56* [23] and *scaA* [44], though additional sequencing primers are required. For large-scale culture-independent studies, smaller fragments containing immunogenic epitopes may be prioritized; however, partial sequences will invariably exclude important phylogenetic signals and reduce congruence with core genome-based phylogeny.

TW-1 and TW-22 genomes had acceptable sequencing coverage (>100×), and no assembly errors were identified using Pilon. Among the genomes examined in this study, TA686 was an outlier in that it possesses >1000 pseudogenes (representing >40% of CDSs), leading to questioning of its assembly accuracy and whether it is accurately placed in phylogenetic analyses. Contaminant reads that map to the host cell genome should be removed before genome assembly, including mitochondrial DNA, which was not depleted with filtration and nuclease treatment.

In conclusion, phylogenetic analysis based on concatenated ScaA and TSA56 sequences offers a substantial improvement over TSA56-based analysis in its ability to reflect genome-scale phylogeny, and future studies should prioritize their sequencing for *O. tsutsugamushi* isolates or clinical specimens if WGS-based methods are not available. ScaA and TSA56 sequences are also valuable to inform antigen selection for vaccine development.

## Figures and Tables

**Table 1:** Summary of complete *Orientia tsutsugamushi* genomes included in this study.

Strain	Country	Year Isolated	Size (bp)	GC Content	Genes	RNA	CDSs	Pseudogenes	GenBank Accession	RefSeq Accession	Reference
Boryong	South Korea	late 1980s ^†^	2,127,051	30.5%	2264	40	2224	533	AM494475	NC_009488	[29]
Ikeda	Japan	1979 ^†^	2,008,987	30.5%	2131	40	2091	423	AP008981	NC_010793 ^§^	[30]
Gilliam *	Burma	1943 ^†^	2,465,012	30.5%	2600	40	2560	566	LS398551	NZ_LS398551	[31]
Karp *	New Guinea	1943 ^†^	2,469,803	30.8%	2525	40	2485	488	LS398548	NZ_LS398548	[31]
Kato *	Japan	1955 ^†^	2,319,449	30.8%	2339	41	2298	431	LS398550	NZ_LS398550	[31]
TA686	Thailand	1963 ^‡^	2,254,553	30.6%	2537	40	2497	1025	LS398549	NZ_LS398549 ^¶^	[31]
UT76	Thailand	2003 ^†^	2,078,193	30.5%	2203	40	2163	451	LS398552	NZ_LS398552	[31]
UT176	Thailand	2004 ^†^	1,932,116	30.2%	1990	41	1949	416	LS398547	NZ_LS398547	[31]
Wuj/2014	China	2014 ^†^	1,972,387	30.5%	2054	40	2014	421	CP044031	NZ_CP044031	unpublished
TW-1	Taiwan	2007 ^†^	2,008,429	30.5%	2067	40	2027	400	CP142421	pending	this study
TW-22	Taiwan	2007 ^†^	2,044,475	30.5%	2192	40	2152	414	CP142420	pending	this study

* Prototype strain, ^†^ clinical isolate, ^‡^ isolated from a wild mammal (*Tupaia glis*), ^§^ reference assembly, ^¶^ record suppressed.

**Table 2:** Congruence between phylogenetic trees based on the quartet similarity measure implemented in R package Quartet (normalized scores are shown) [72].

	Maximum Likelihood	Neighbor-Joining
	core	core
core	1.0000	1.0000
TSA56	0.5910	0.5910
ScaA	0.0818	0.0818
ScaC	0.3180	0.4000
ScaD	0.1680	0.2450
ScaE	0.2640	0.2270
ScaA + TSA56	0.9270	0.9640
ScaC + TSA56	0.5910	0.5640
ScaD + TSA56	0.6730	0.6450
ScaE + TSA56	0.6360	0.6360

## Data Availability

Sequence data are available in the NCBI Sequence Read Archive under BioProject Accession Number PRJNA987430.

## Supplementary Materials

The following supporting information can be downloaded at: https://www.mdpi.com/article/10.3390/pathogens13040299/s1, Figure S1: Growth curves, Figure S2: Summary of filtered HiFi reads used for de novo assembly, Figure S3: Circular chromosome of *Orientia tsutsugamushi* strain TW-1, Figure S4: Circular chromosome of *Orientia tsutsugamushi* strain TW-22, Table S1: Pairwise amino acid alignments of *Orientia tsutsugamushi* surface antigens, Table S2: Summary of NCBI locus tags for surface antigens, Figure S5: Maximum likelihood phylogenetic trees based on individual amino acid sequences, Figure S6: Neighbor-joining phylogenetic trees based on individual amino acid sequences, Figure S7: Maximum likelihood phylogenetic trees based on concatenated amino acid sequences, Figure S8: Neighbor-joining phylogenetic trees based on concatenated amino acid sequences.
