# Supplementary material for: Concatenated ScaA and TSA56 Surface Antigen Sequences Reflect Genome-Scale Phylogeny of Orientia tsutsugamushi: An Analysis Including Two Genomes from Taiwan
Source: Pathogens. 2024 Apr 3;13(4):299. doi: 10.3390/pathogens13040299 (PMC11054523; doi:10.3390/pathogens13040299)
Supplement: Supplementary file 1 [file pathogens-13-00299-s001.zip › pathogens-2931530-supplementary.pdf]

Supplementary Materials

Concatenated ScaA and TSA56 Surface Antigen Sequences Reflect Genome-Scale Phylogeny of *Orientia tsutsugamushi*: An Analysis Including Two Genomes from Taiwan

Nicholas T. Minahan [1], Tsai-Ying Yen [2], Yue-Liang Leon Guo [1,3], Pei-Yun Shu [2] and Kun-Hsien Tsai [1,4,*]

[1] Institute of Environmental and Occupational Health Sciences, College of Public Health, National Taiwan University, Taipei 100025, Taiwan; f05841021@ntu.edu.tw (N.T.M.); leonguo@ntu.edu.tw (Y.-L.L.G.)

[2] Centers for Diagnostics and Vaccine Development, Centers for Disease Control, Ministry of Health and Welfare, Taipei 115210, Taiwan; yenty@cdc.gov.tw (T.-Y.Y.); cdcpyshu@gmail.com (P.-Y.S.)

[3] Department of Environmental and Occupational Medicine, National Taiwan University (NTU) College of Medicine and NTU Hospital, Taipei 100025, Taiwan

[4] Global Health Program, College of Public Health, National Taiwan University, Taipei 100025, Taiwan

* Correspondence: kunhtsai@ntu.edu.tw

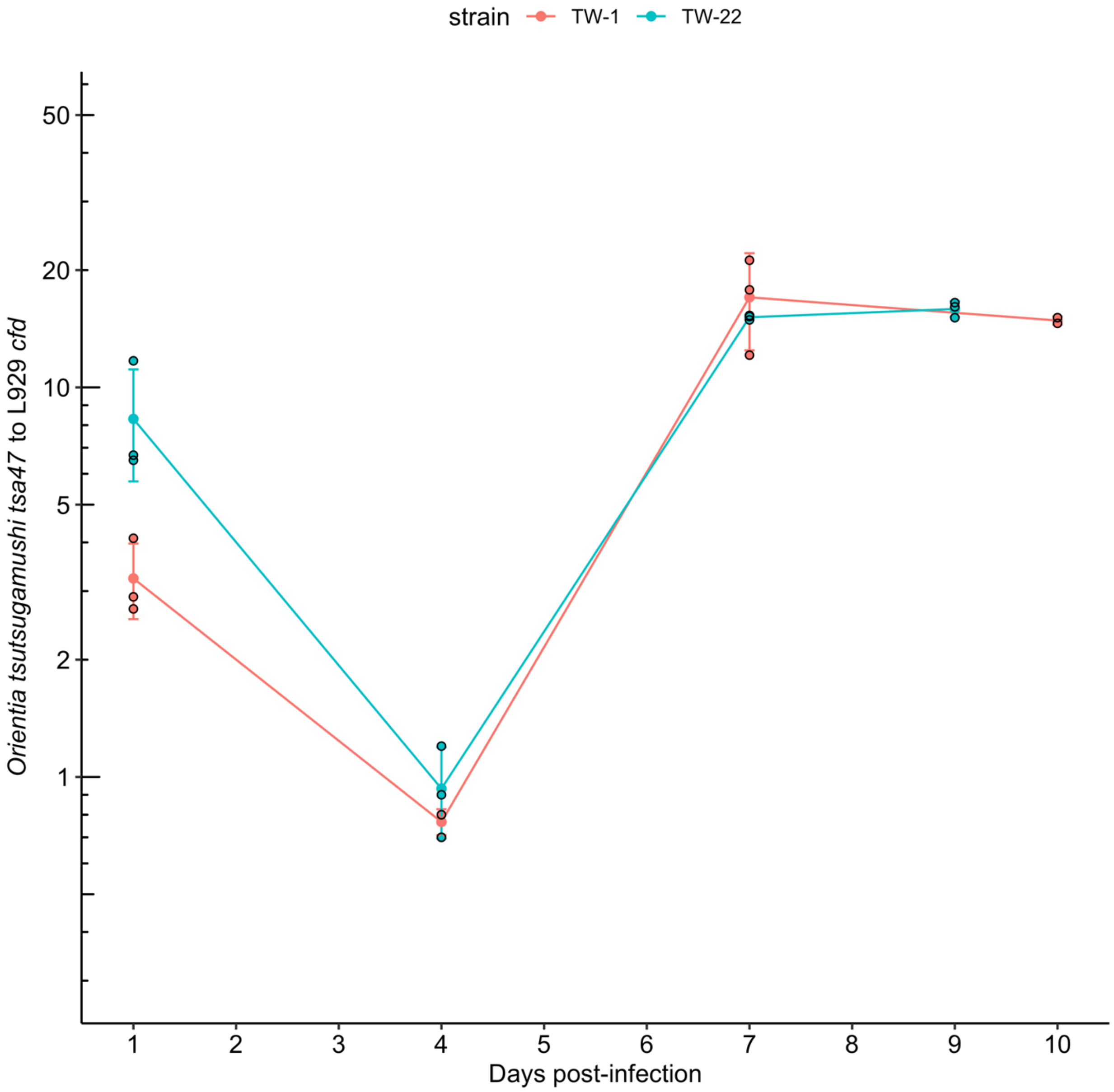

Figure S1. Growth curves. Mean ratio of *Orientia tsutsugamushi* (OT) to L929 cells in the supernatant of cultures (passage 8) of strains TW-1 and TW-22 based on single-copy targets. Each point represents mean values of triplicate measurements for an individual 75 cm^2 (T75) flask. Colored dots represent mean values, and error bars represent standard deviation.

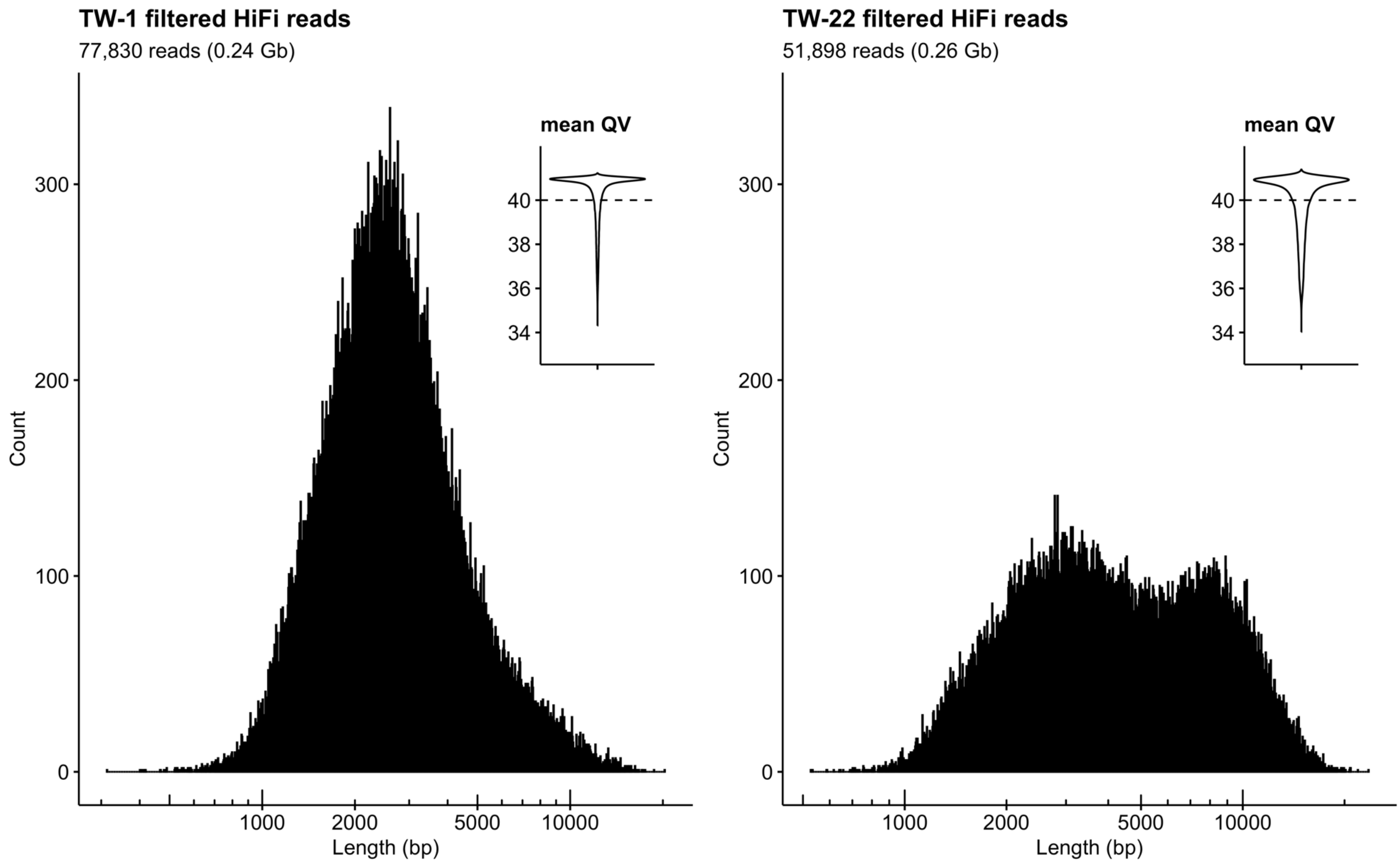

Figure S2. Summary of filtered HiFi reads used for de novo assembly. Quality values (QVs) were capped at 41 (representing <1 in 10,000 probability of error) using Biopython 1.81, and mean QVs were calculated using qckitfastq 1.16.0.

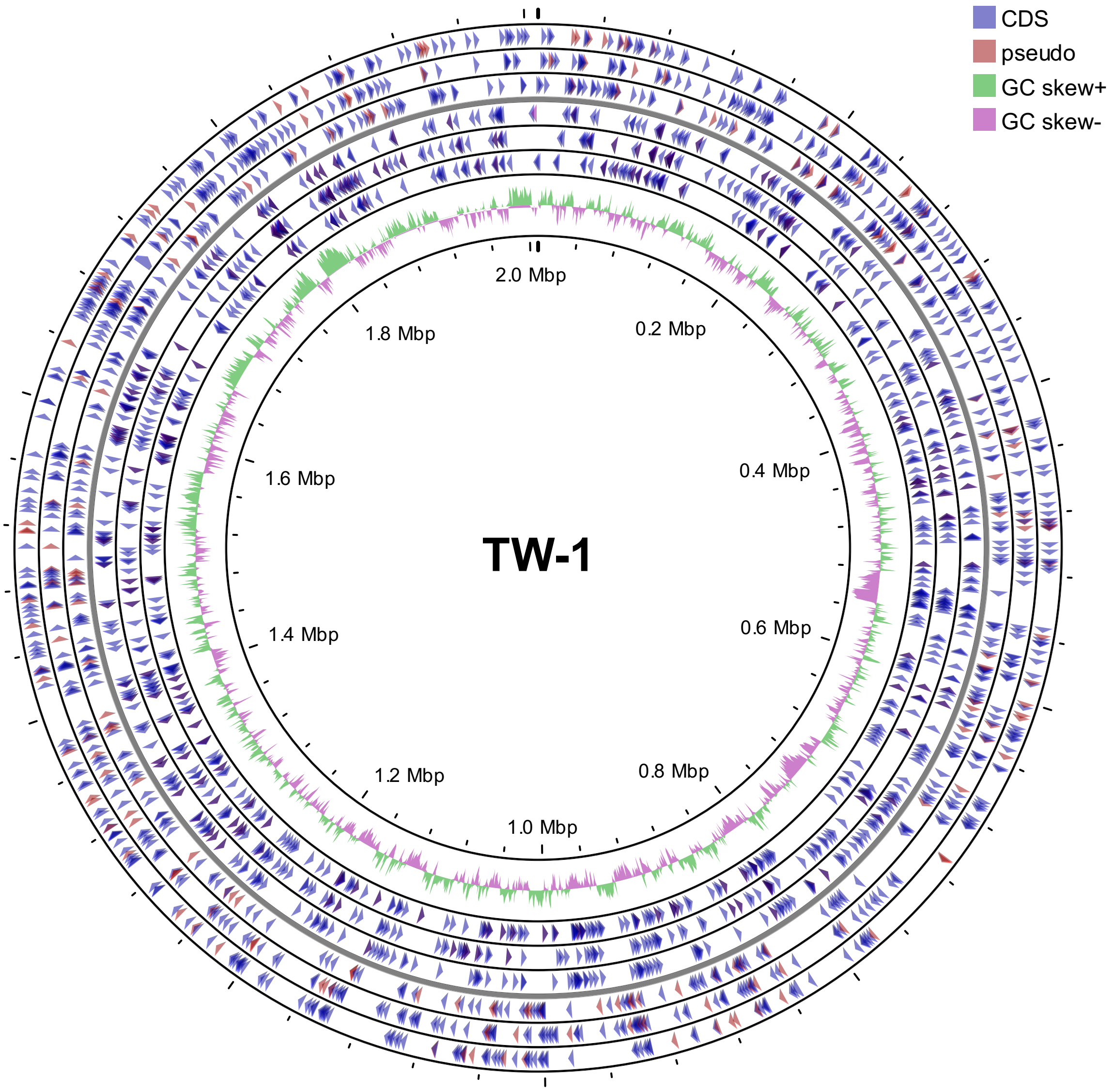

Figure S3. Circular chromosome of *Orientia tsutsugamushi* strain TW-1. CDS = protein-coding sequence; pseudo = pseudogene.

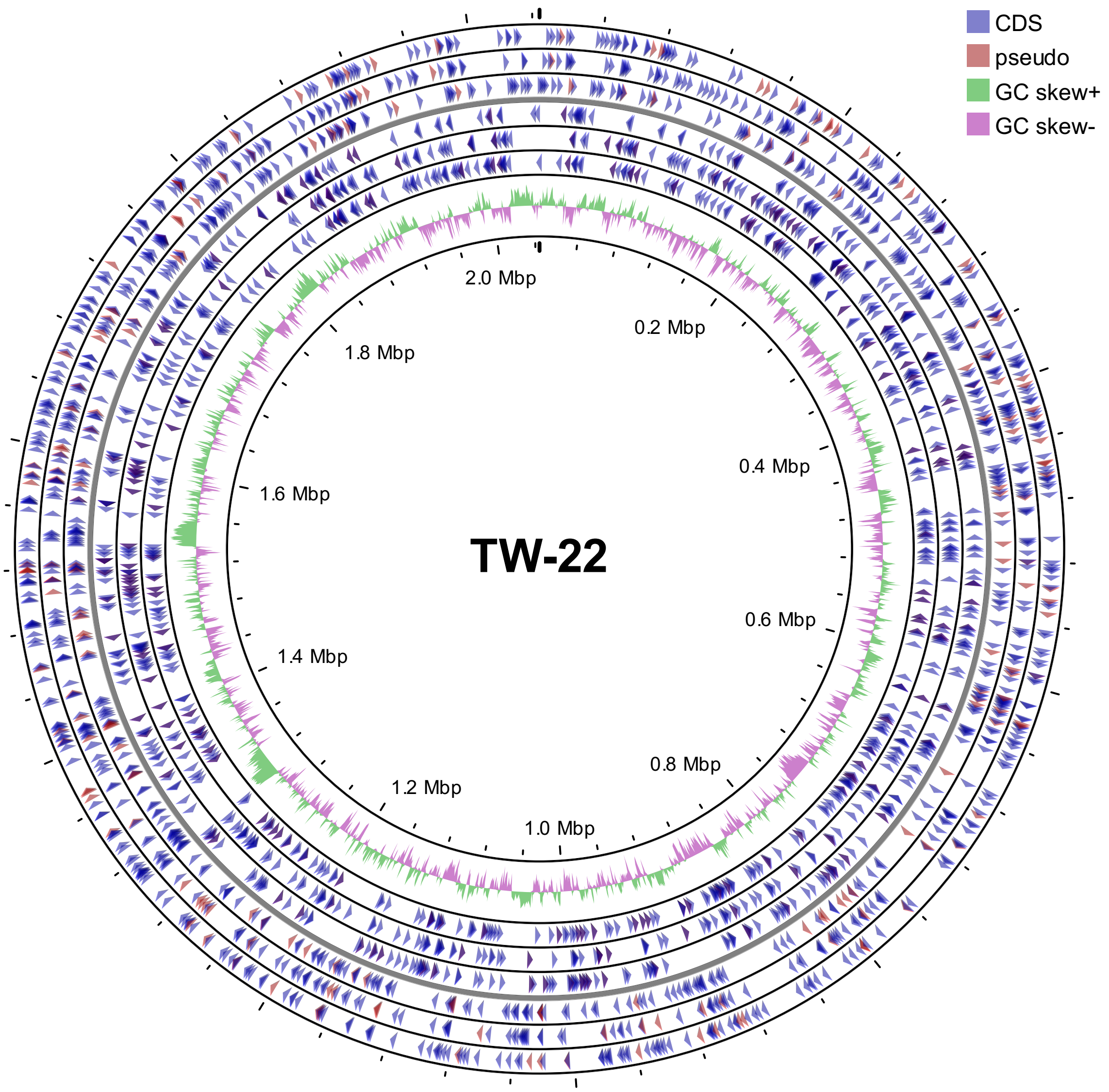

Figure S4. Circular chromosome of *Orientia tsutsugamushi* strain TW-22. CDS = protein-coding sequence; pseudo = pseudogene.

Table S1. Pairwise amino acid alignments of *Orientia tsutsugamushi* surface antigens.

Strain	TSA56	ScaA	ScaB	ScaC	ScaD	ScaE	ScaF
Boryong	**532 aa (reference)**	**1461 aa (reference)**	649 aa; **2 copies**	**526 aa (reference)**	**998 aa (reference)**	**760 aa (reference)**	absent
Ikeda	516 aa (532 aa; 18,540 bits; 72.74% id)	1508 aa (1532 aa; 57,020 bits; 75.52% id)	absent	520 aa (526 aa; 23,620 bits; 88.21% id)	834 aa (998 aa; 30,700 bits; 69.34% id)	753 aa (778 aa; 28,200 bits; 77.25% id)	absent
Gilliam	524 aa (532 aa; 21,860 bits; 82.89% id)	1518 aa (1529 aa; 58,610 bits; 77.17% id)	absent	517 aa (526 aa; 23,590 bits; 88.78% id)	745 aa (998 aa; 26,620 bits; 63.53% id)	757 aa (774 aa; 28,280 bits; 76.74% id)	absent
Karp	532 aa (534 aa; 22,650 bits; 84.08% id)	1514 aa (1529 aa; 56,810 bits; 75.67% id)	absent	517 aa (526 aa; 23,380 bits; 87.83% id)	988 aa (1000 aa; 35,330 bits; 73.70% id)	755 aa (772 aa; 26,510 bits; 72.54% id)	645 aa (645 aa; 32,750 bits; 99.38% id)
Kato	529 aa (544 aa; 17,630 bits; 68.93% id)	1447 aa (1470 aa; 59,320 bits; 79.52% id)	absent	520 aa (526 aa; 23,780 bits; 89.54% id)	872 aa (998 aa; 34,060 bits; 73.35% id)	747 aa (775 aa; 25,470 bits; 71.10% id)	absent
TA686	532 aa (536 aa; 19,540 bits; 75.37% id)	1446 aa (1464 aa; 57,020 bits; 77.66% id)	absent	517 aa (526 aa; 23,570 bits; 88.78% id)	777 aa (998 aa; 28,500 bits; 66.03% id)	756 aa (774 aa; 26,720 bits; 73.00% id)	**645 aa (reference)**
UT76	536 aa (543 aa; 21,380 bits; 81.22% id)	1526 aa (1531 aa; 57,350 bits; 75.11% id)	absent	517 aa (526 aa; 23,360 bits; 87.26% id)	1035 aa (1036 aa; 37,150 bits; 74.90% id)	751 aa (772 aa; 26,600 bits; 72.80% id)	absent
UT176	533 aa (534 aa; 22,630 bits; 83.52% id)	1508 aa (1533 aa; 56,530 bits; 75.21% id)	absent	520 aa (526 aa; 23,830 bits; 88.97% id)	872 aa (998 aa; 33,260 bits; 71.94% id)	747 aa (765 aa; 26,690 bits; 73.33% id)	absent
Wuj/2014	535 aa (542 aa; 21,570 bits; 81.00% id)	1524 aa (1544 aa; 58,630 bits; 77.07% id)	absent	523 aa† (526 aa; 23,630 bits; 88.02% id)	988 aa (1002 aa; 37,420 bits; 77.15% id)	749 aa (774 aa; 26,480 bits; 73.51% id)	absent
TW-1	535 aa (542 aa; 21,480 bits; 80.63% id)	1531 aa (1547 aa; 56,330 bits; 74.53% id)	absent	523 aa† (526 aa; 23,630 bits; 88.02% id)	941 aa (999 aa; 36,700 bits; 76.38% id)	749 aa (774 aa; 26,420 bits; 73.39% id)	absent
TW-22	524 aa (539 aa; 18,160 bits; 71.24% id)	1507 aa (1525 aa; 57,870 bits; 79.38% id)	absent	520 aa (526 aa; 23,900 bits; 89.16% id)	807 aa (998 aa; 29,650 bits; 68.04% id)	748 aa (772 aa; 26,180 bits; 72.80% id)	absent
	68.93-99.63% identity	*71.32-89.16% identity*	*NA*	*86.35-100% identity*	*63.53-95.41% identity*	*71.10-99.73% identity*	*NA*

aa = amino acids; id = identity; † = identical

Table S2. Summary of NCBI locus tags for surface antigens.

	**Boryong** (AM494475)	**Ikeda** (AP008981)	**Gilliam** (LS398551)	**Karp** (LS398548)	**Kato** (LS398550)	**TA686** (LS398549)	**UT76** (LS398552)	**UT176** (LS398547)	**Wuj/2014** (CP044031)	**TW-1** (CP142421)	**TW-22** (CP142420)
**TSA56**	OTBS_ 0602	OTT_ 0945	GILLIAM_ 02257	KARP_ 02001	KATO_ 00907	TA686_ 02342	UT76HP_ 01865	UT176_ 00941	F0363_ 02480	QU601_ 001572	QU600_ 000493
**ScaA**	OTBS_ 0102	OTT_ 1215	GILLIAM_ 01513	KARP_ 00798	KATO_ 00211	TA686_ 00130	UT76HP_ 00328	UT176_ 00529	F0363_ 06280	QU601_ 000243	QU600_ 001479
**ScaB**	OTBS_ 0864 OTBS_ 2137										
**ScaC**	OTBS_ 1686	OTT_ 0460	GILLIAM_ 01556	KARP_ 01628	KATO_ 01770	TA686_ 01405	UT76HP_ 01782	UT176_ 01268	F0363_ 02065	QU601_ 001499	QU600_ 000816
**ScaD**	OTBS_ 1913	OTT_ 1741	GILLIAM_ 01380	KARP_ 00576	KATO_ 00608	TA686_ 00458	UT76HP_ 02170	UT176_ 00383	F0363_ 04340	QU601_ 001941	QU600_ 001734
**ScaE**	OTBS_ 2126	OTT_ 1766	GILLIAM_ 00783	KARP_ 00606	KATO_ 00640	TA686_ 00866	UT76HP_ 02139	UT176_ 00408	F0363_ 04165	QU601_ 001910	QU600_ 001689
**ScaF**				KARP_ 01610		TA686_ 01679					

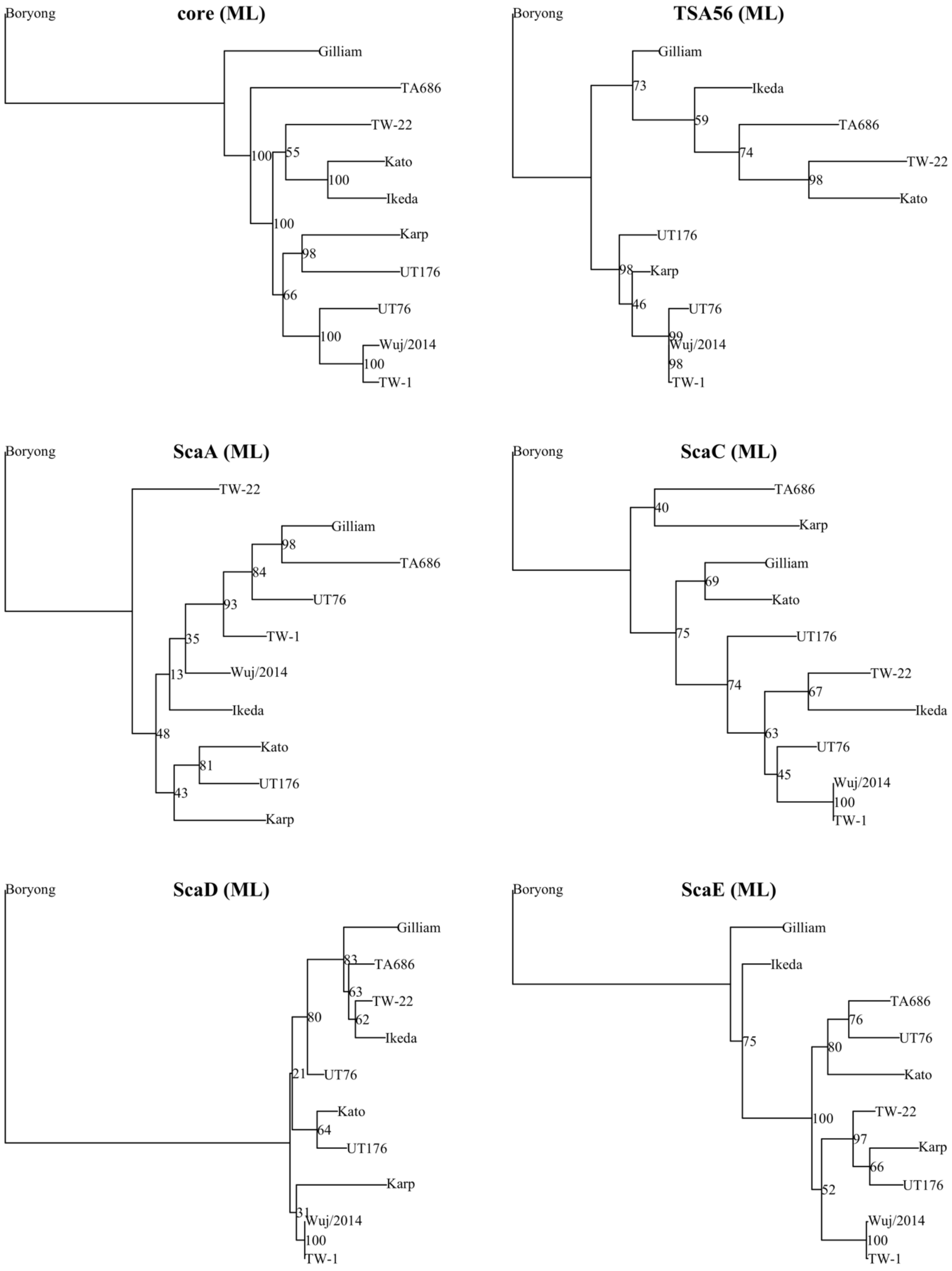

Figure S5. Maximum likelihood phylogenetic trees based on individual amino acid sequences.

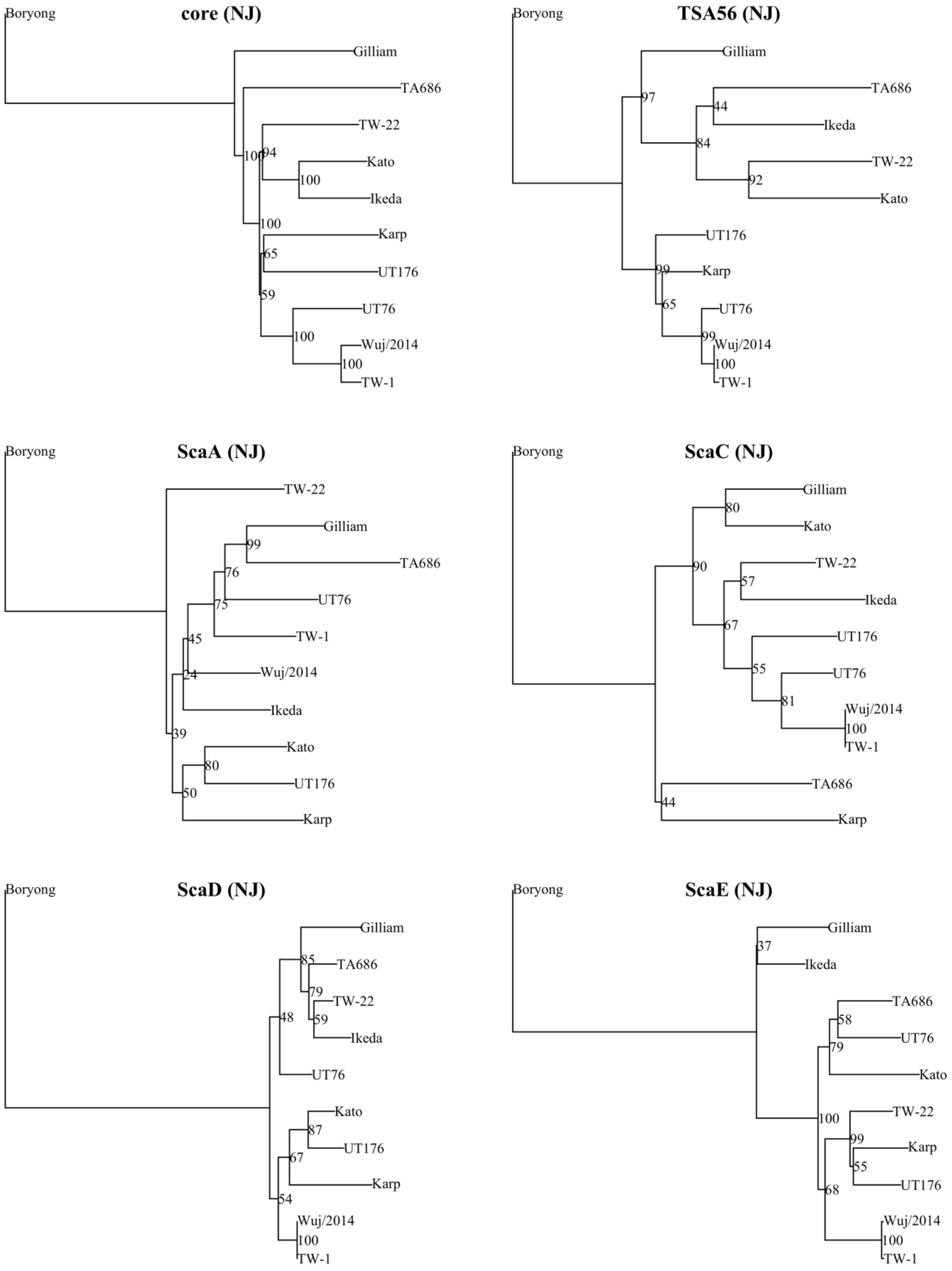

Figure S6. Neighbor-joining phylogenetic trees based on individual amino acid sequences.

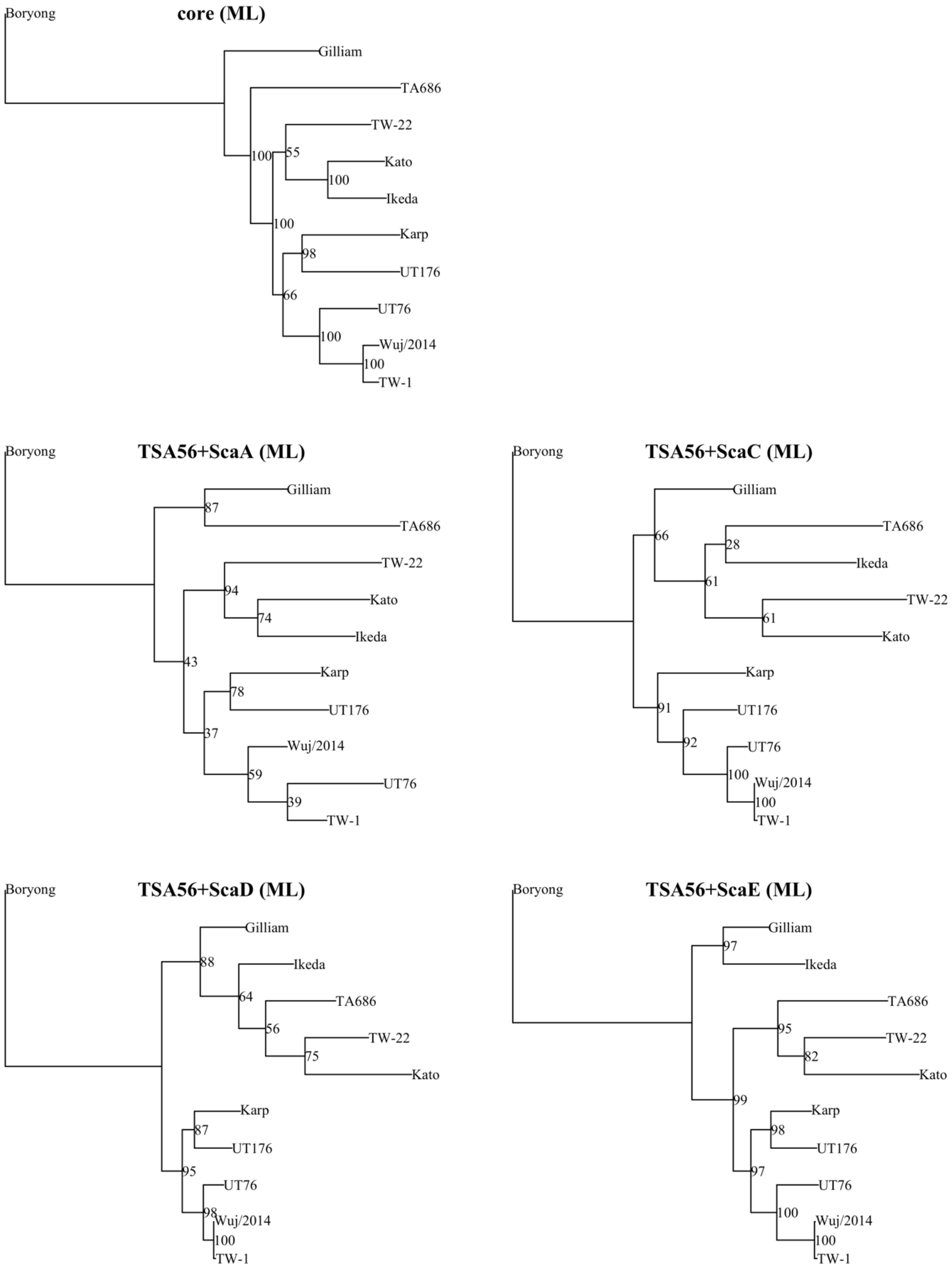

Figure S7. Maximum likelihood phylogenetic trees based on concatenated amino acid sequences.

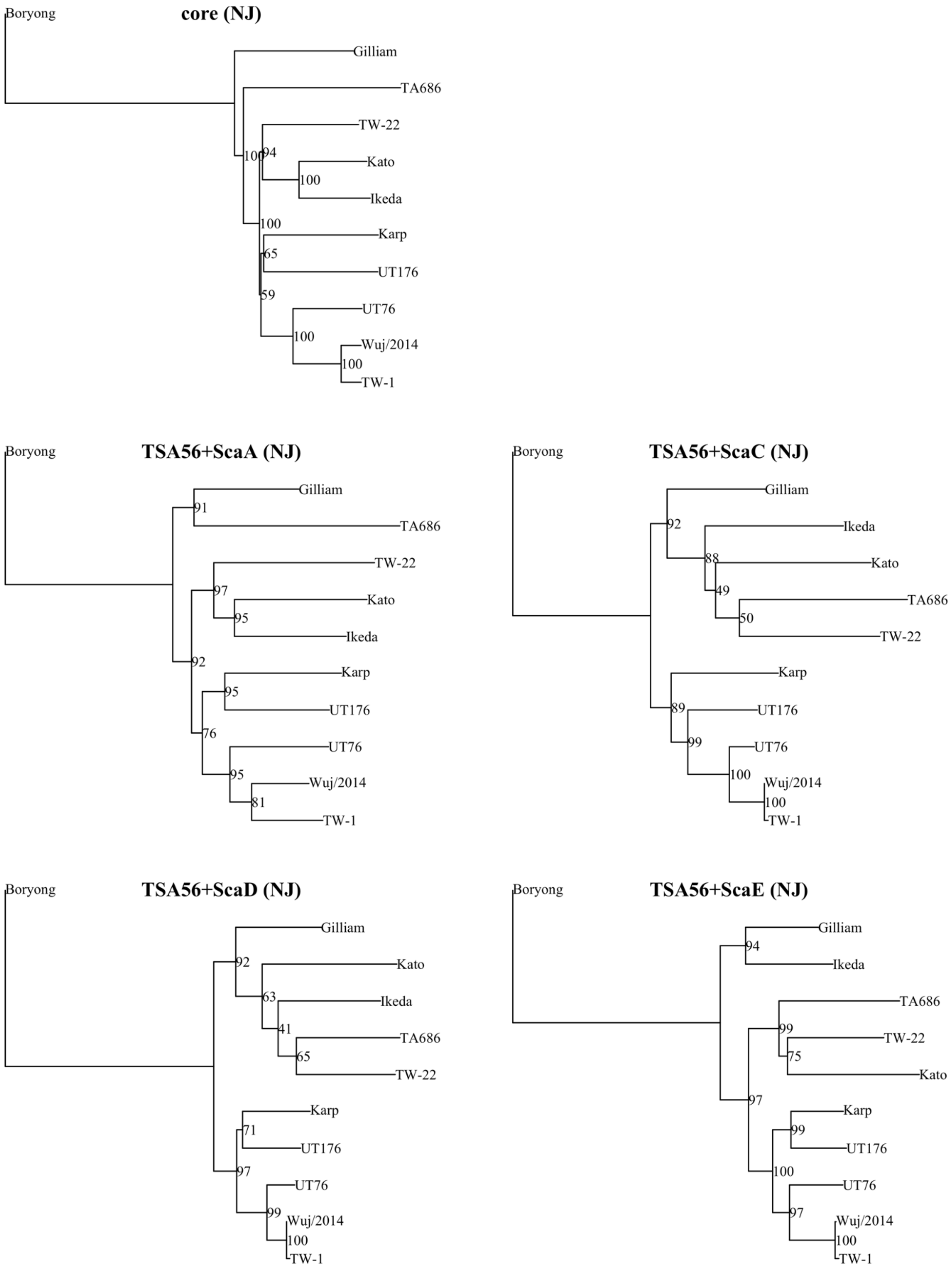

Figure S8. Neighbor-joining phylogenetic trees based on concatenated amino acid sequences.
